# Does dance therapy benefit the improvement of blood pressure and blood lipid in patients with hypertension? A systematic review and meta-analysis

**DOI:** 10.3389/fcvm.2024.1421124

**Published:** 2024-10-24

**Authors:** Jialin Wang, Yikun Yin, Zhengze Yu, Qihan Lin, Yongsheng Liu

**Affiliations:** ^1^ Institute of Sports Medicine and Health, Chengdu Sport University, Chengdu, China; ^2^ School of Sport Human Science, Beijing Sport University, Beijing, China; ^3^ College of Physical Education and Health, Guangxi Normal University, Guilin, China; ^4^ College of Physical Education and Health, Longyan University, Longyan, China; ^5^ School of Physical Education, Jining University, Jining, China

**Keywords:** dance therapy, hypertension, blood pressure, blood lipid, meta-analysis

## Abstract

**Objective:**

Hypertension is a risk factor of cardiovascular disease. Dance, a type of aerobic exercise, is beneficial as a therapy in reducing blood pressure. This study aimed to systematically review the therapeutic effectiveness of dance therapy (DT) on blood pressure and blood lipid of patients with hypertension.

**Methods:**

Searching CNKI, VIP, Wan Fang Databases, CBM, PubMed, EBSCO (MEDLINE), Cochrane Library, and Web of Science to collect randomized controlled trials (RCTs) about dance therapy in the treatment of patients with hypertension according to the inclusion and exclusion criteria, with the search time ranged from the date of database construction to January 2024. The Cochrane risk-of-bias tool and PEDro were used to evaluate the risk of included trials. The meta-analysis was implemented by using RevMan 5.4 and Stata 12.0 software.

**Results:**

A total of 983 patients were included in 11 randomized controlled trials. According to the meta-analysis, compared with the control group, Dance Therapy effectively reduced systolic blood pressure (SBP) [MD = −7.45, 95% CI (−8.50, −6.39), *p* < 0.0001] and diastolic blood pressure (DBP) [MD = −2.95, 95% CI (−3.78, −2.13), *p* < 0.0001], and it increased high-density lipoprotein cholesterol (HDL-C) [MD = 0.20, 95% CI (−0.02, 0.42), *p* < 0.0001]. The subgroup analysis results showed that the treatment efficacy was more excellent with the frequency more than 3 times per week, the cycle less than 12 weeks, and the duration less than 60 min every time.

**Conclusion:**

The results indicates that SBP, DBP, and HDL-C in hypertensive patients have been effectively improved after dance therapy intervention. In addition, it is recommended to implement dance therapy for hypertensive patients with a treatment cycle of 12 weeks, and treat at least 3 times a week, with each treatment duration controlled within 60 min.

**Systematic Review Registration:**

[http://www.crd.york.ac.uk/PROSPERO], identifier [CRD42024500807].

## 1 Introduction

Hypertension is the most prevalent risk factor of cardiovascular disease. About 9.4 million individuals worldwide succumb to cardiovascular complications or other complications caused by hypertension every year, accounting for about one fifth of the total deaths worldwide (1). Globally, over 1.3 billion individuals suffer from hypertension (2, 3). It is estimated that the proportion of patients with hypertension will rise to 29%, about 1.5 billion people, in 2025 (4). It is a very serious challenge for the global healthcare system. Drug therapy to reduce blood pressure entail higher costs, increased side effects and poor patient compliance (5). In 2020, the World Health Organization proposed that physical activity in patients with hypertension can improve the incidence of cardiovascular disease, reduce mortality, and improve the quality of life (6). Effective physical exercise and a healthy lifestyle constitute pivotal elements in cardiovascular disease prevention and blood pressure management. Aerobic physical exercises, such as dancing, walking, jogging and swimming, no less than 5 days per week and no less than 30 min per day are conducive to reducing blood pressure (7–9). Low salt diet and reducing the intake of red meat, sugar and trans-fat are also conducive to blood pressure management (10, 11).

Dance, as a long-term, strong dynamic, large muscle group participation and communal aerobic exercise, plays a positive role in reducing blood pressure, improving cardiopulmonary function, enhancing muscle strength and physical flexibility, alleviating stress and anxiety, and promoting healthy lifestyle habits (12). Dance therapy (DT), which is derived from dance, is a therapeutic method that combines personal emotion, cognition, physicality, and social interaction through dance to regulate emotions, manage illnesses, and establish physical and mental balance (13).

Different studies employed different exercise methods, durations, frequencies, and cycles of DT interventions, leading to different results in blood pressure improvement among patients with hypertension. In this systematic review and meta-analysis, we integrated the results of recent randomized controlled studies (RCTs) on dance therapy interventions for hypertension, and conducted a systematic, objective and quantitative statistical analysis. This study aims to investigate the potential benefits of DT on blood pressure and blood lipid levels in patients with hypertension, and to offer the latest evidence-based guidance for the clinical practice of DT.

## 2 Materials and methods

### 2.1 Retrieval strategy

The systematic review and meta-analysis were planned and implemented in accordance with the Preferred Reporting Items for Systematic Reviews and Meta-Analyses (PRISMA) guidelines (14). The review project was registered on the international prospective register of systematic reviews (http://www.crd.york.ac.uk/PROSPERO) with a registration number CRD42024500807.

We searched multiple databases including CNKI, VIP, Wanfang Data, China Biology Medicine disc (CBM), PubMed, EBSCO (MEDLINE), Cochrane Library and Web of Science. We limited the search period from the dates of databases established to January 2024. The final search was conducted on January 10, 2024. The search terms comprised “dance”, “dancing exercises”, “dance training”, “aerobic dance”, “hypertension”, “blood pressure”, “high blood pressure” and “hypertension”. In order to obtain the most complete RCTs related to the treatment of DT in hypertensive patients, we also traced the references of the included literature to supplement the retrieval of relevant literature. The complete search strategies for each database are provided in Supplementary Table S1.

### 2.2 Literature inclusion, exclusion criteria and outcome indicator

Inclusion criteria: ① The study design should be a randomized controlled trial about DT in the treatment of patients with hypertension. ② participants included in the study were patients with primary hypertension (regardless of gender, age, race and nationality). Diagnosis of hypertension adhered to international diagnostic criteria, such as systolic blood pressure (SBP) ≥ 140 mmHg (1 mmHg = 0.133 kPa) and diastolic blood pressure (DBP) ≥ 90 mmHg. Studies involving participants with secondary hypertension or other cardiovascular diseases were excluded. ③ The main intervention method in the included studies should be dance training alone or in combination with other interventions. The intervention method of the controls should be any other treatment method, including conventional medication treatment, usual care, health education, other exercise therapy or no treatment. ④ The included studies should have complete original data for direct or indirect extraction for analysis. ⑤ The included should be published in either Chinese or English.

Exclusion criteria: Non-Chinese and English literature, repeatedly published literature, literature from which data extraction and retrieval of the original text were not effectively feasible, non-journal-published research, animal studies, cross-sectional studies, non-clinical experimental research, and experimental research without intervention design were excluded.

Outcome indicators included systolic blood pressure (SBP), diastolic blood pressure (DBP), total cholesterol (TC), triglycerides (TG), low-density lipoprotein cholesterol (LDL-C), high-density lipoprotein cholesterol (HDL-C), Body Mass Index(BMI) and Resting heart rate (RHR).

### 2.3 Literature screening and information extraction

Literature screening and data extraction were conducted by two independent reviewers (Jialin Wang and Yongsheng Liu). Any disagreements encountered during cross-checking will be resolved through discussion or negotiation with the third reviewer (Qihan Lin). During literature screening, we reviewed the article titles first. Subsequently, we excluded obviously irrelevant literature and proceeded to review the abstracts and full texts to determine inclusion. If necessary, we will contact the original study author via email or phone to obtain the undetermined information.

Data extraction encompassed the following aspects: ① Basic information of included studies, including the first author, the publication year, the sample size, the intervention measure, the intervention frequency, the intervention duration and the evaluation indicator; ②Specific intervention details and the follow-up duration; ③ Key elements of bias risk assessment; ④ Outcome indicators and measurement data of interest.

### 2.4 Assessment of risk of bias and study quality

Two independent reviewers (Jialin Wang and Yikun Yin). conducted the assessment of risk of bias and study quality. If the evaluation results of the two reviewers are inconsistent, consultation with the third independent reviewer (Zhengze Yu) is required. Risk of bias was assessed using Cochrane Collaboration Tool 5.1.0 for randomized controlled trials. Risk of bias figures were generated using RevMan5.4. The Physiotherapy Evidence Database (PEDro) scale was used to assess the risk of bias and methodological quality of included studies (15). The PEDro scale scores study on a scale of 0–10. The evaluation criteria state that 1 point is awarded for each criterion met from items 2–11, with a maximum score of 10 points. Studies scoring ≥6 are considered high quality, those scoring 4–5 are considered moderate quality, and those scoring ≤3 are considered low quality (16).

### 2.5 Statistical analysis

Statistical analysis of data extracted from the included literature was performed using RevMan5.4, a meta-analysis software provided by the Cochrane Collaboration. Continuous variable outcome indicators from the included literature were statistically analyzed using mean difference (MD) and 95% confidence interval (CI). If the same outcome indicator is extracted with identical measurement methods and units, the weighted mean difference (WMD), the weighted mean difference (WMD) is selected as the effect size indices. Heterogeneity among the results of the included studies was assessed using *p*-value and *I*^2^ quantification. No heterogeneity was observed among studies when *p* ≥ 0.10, whereas heterogeneity existed when *p* < 0.10. *I*^2^ represents the level of heterogeneity between studies. If *I*^2^ < 50%, it indicates that there was slight heterogeneity between the studies, and the fixed effect model was used for analysis. If *I*^2^ ≥ 50%, there was heterogeneity in the study, and the random effect model was used for analysis (17). The *α* value was set at 0.05.Additionally, Stata 12.0 software was employed to perform publication bias analysis and sensitivity analysis using Begg's test for studies with more than 5 included outcome indicators. The threshold for statistical significance was set at *p* < 0.05 (18).

## 3 Result

### 3.1 Study selection

**Figure 1:**
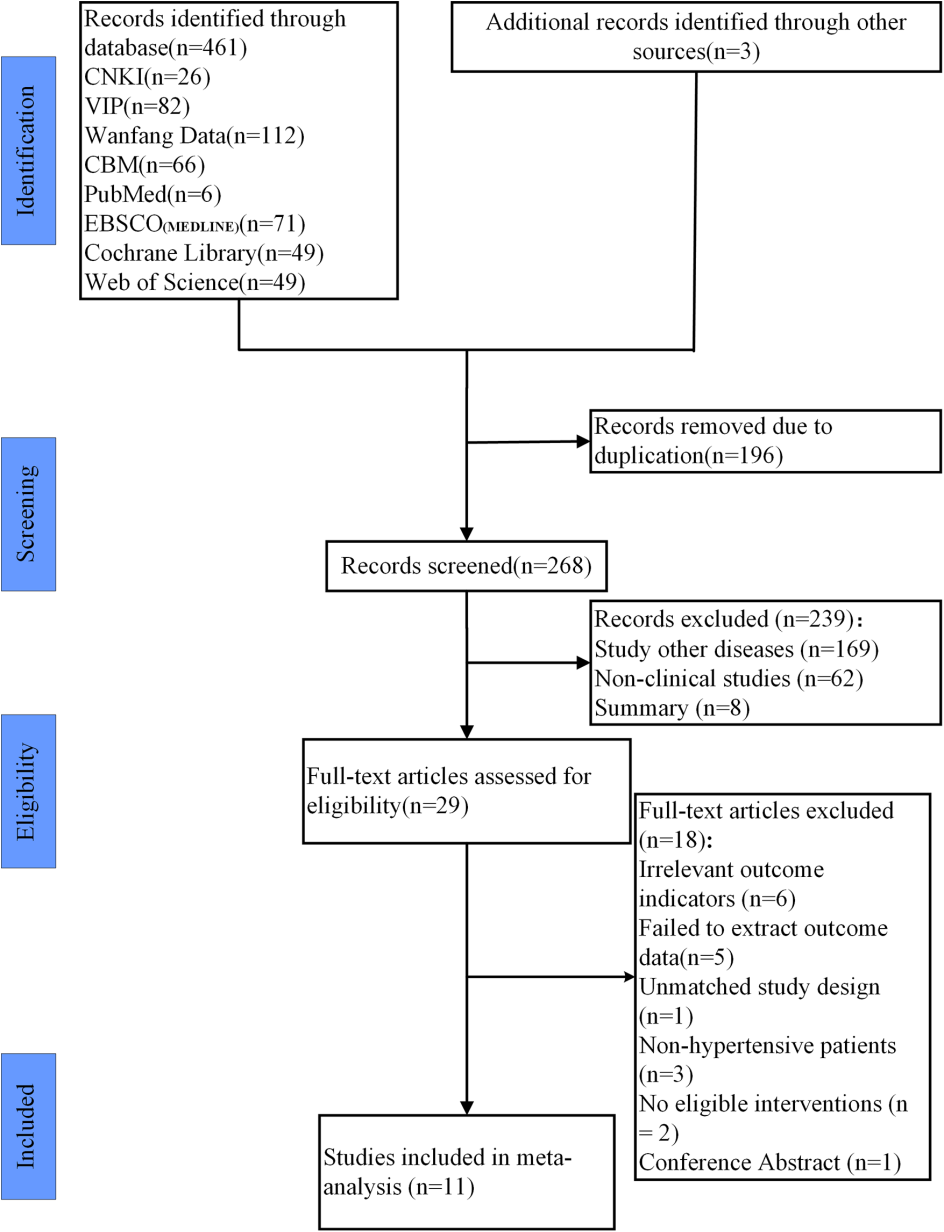
Study selection represented by PRISMA flowchart. Note: After screening the literature, we reviewed the references and found an additional 3 studies.

461 studies were retrieved from the databases, and 3 studies were found through reference tracing. Endnote X9 was used to manage retrieved literature and remove duplicates, resulting in 268 studies remaining. After reading the title and abstract, 29 studies were selected. After reading the full texts, 18 studies were excluded due to not meeting the inclusion and exclusion criteria. Finally, 11 studies were included (7, 12, 19–27). The process of study inclusion is shown in Figure 1.

### 3.2 Study characteristics

11 included studies involved a total of 983 participants (7, 12, 19–27), with intervention durations ranging from 4 to 24 weeks. Participants’ average age ranged from 31.4 to 72.7 years, and sample sizes ranged from 44 to 120 participants. 9 of the included studies, accounting for 90%, were published in the past decade (2014–2023). Among the included studies, 4 studies utilized square dance as the intervention for the experimental group (22–24, 27), 2 studies used aerobic dance (7, 20), and 6 studies employed health education as the intervention for the control group (12, 19, 21, 23–25). Additionally, 3 studies had no exercise intervention (22, 26, 27), while 2 studies conducted drug therapy (7, 20). Among the included studies, 7 had a 12-week exercise cycle, 3 had a cycle of less than 12 weeks, with an average cycle duration of 10.4 weeks. The average exercise frequency was 3.5 times per week. 6 studies had exercise durations of 60 min or more, while 4 had durations less than 60 min. The details of study characteristics are shown in Table 1 and Supplementary Table S2.

**Table 1:** Description of studies included in the meta-analysis.

Author and year of publication	Year		Sample size		Intervention		Dosage	Outcome
	T	C	T	C	T	C		
Yaping et al. (23)	62.0 ± 7.7	65.9 ± 6.0	22	26	Square dance	Health education	60 min each time; three times a week for twelve weeks	①②③④⑤⑥⑦
Yaping et al. (24)	62.0 ± 7.7	65.9 ± 6.0	22	26	Square dance	Health education	60 min each time; three times a week for twelve weeks	①②⑧
Yanbing et al. (26)	62. 00 ± 5. 07	59. 38 ± 4. 51	22	22	Taiji soft ball dance	No exercise intervention	90 min each time; four times a week for twelve weeks	①②③④⑤⑥⑦
Yi et al. (22)	40–59	40–59	55	52	Square dance	No exercise intervention	60–120 min each time; five-seven times a week for twelve weeks	①②③④⑤⑥
Yan et al. (27)	59.54 ± 5.85	60.78 ± 7.21	48	47	Square dance	No exercise intervention	60–120 min each time; five times a week for twelve weeks	①②⑦⑧
Aweto et al. (19)	44.1 ± 12.7	46.4 ± 11.6	23	15	Dance therapy	Health education	50 min each time; four times a week for four weeks	①②⑧
Kaholokula et al. (21)	55 ± 10	55 ± 12	25	23	Hula	Health education	60 min each time; two times a week for twelve weeks	①②
Maruf et al. (20)	50.80 ± 8.31	54.75 ± 8.56	60	60	Aerobic dance	Drug therapy	45 min each time; there times a week for twelve weeks	①②③④⑤⑥
Serrano-Guzmán et al. (12)	69.07 ± 4.41	69.48 ± 3.22	27	25	Dance therapy	Health education	50 min each time; there times a week for teight weeks	①②⑦
Maruf et al. (7)	50.80 ± 8.31	54.75 ± 8.56	60	60	Aerobic dance	Drug therapy	45 min each time; there times a week for eight weeks	①②
Kaholokula et al. (25)	58.1 ± 13.7	57.9 ± 12.6	131	132	Hula	Health education	60 min each time; four times a week for twenty-four weeks	①②

Notes: T, experimental groups; C, control groups; ①Systolic blood pressure (SBP); Diastolic blood pressure (DBP); ③Total cholesterol (TC); ④Triglyceride (TG); ⑤Low density lipoprotein cholesterol (LDL-C); ⑥High-density lipoprotein cholesterol (HDL-C); ⑦Body Mass Index(BMI); ⑧Resting heart rate (RHR); “-” indicates not mentioned. W, week; m, month; y, year.

### 3.3 Study quality

**Figure 2:**
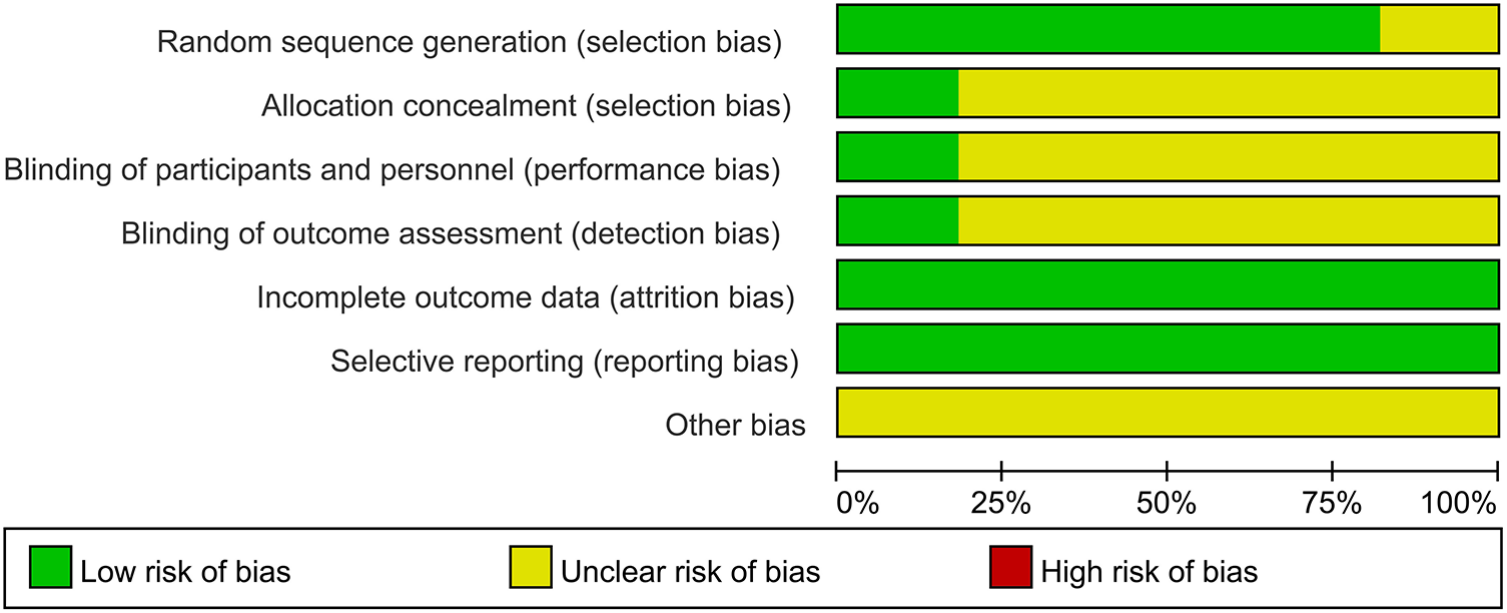
Resume of results of risk of bias assessment.

**Figure 3:**
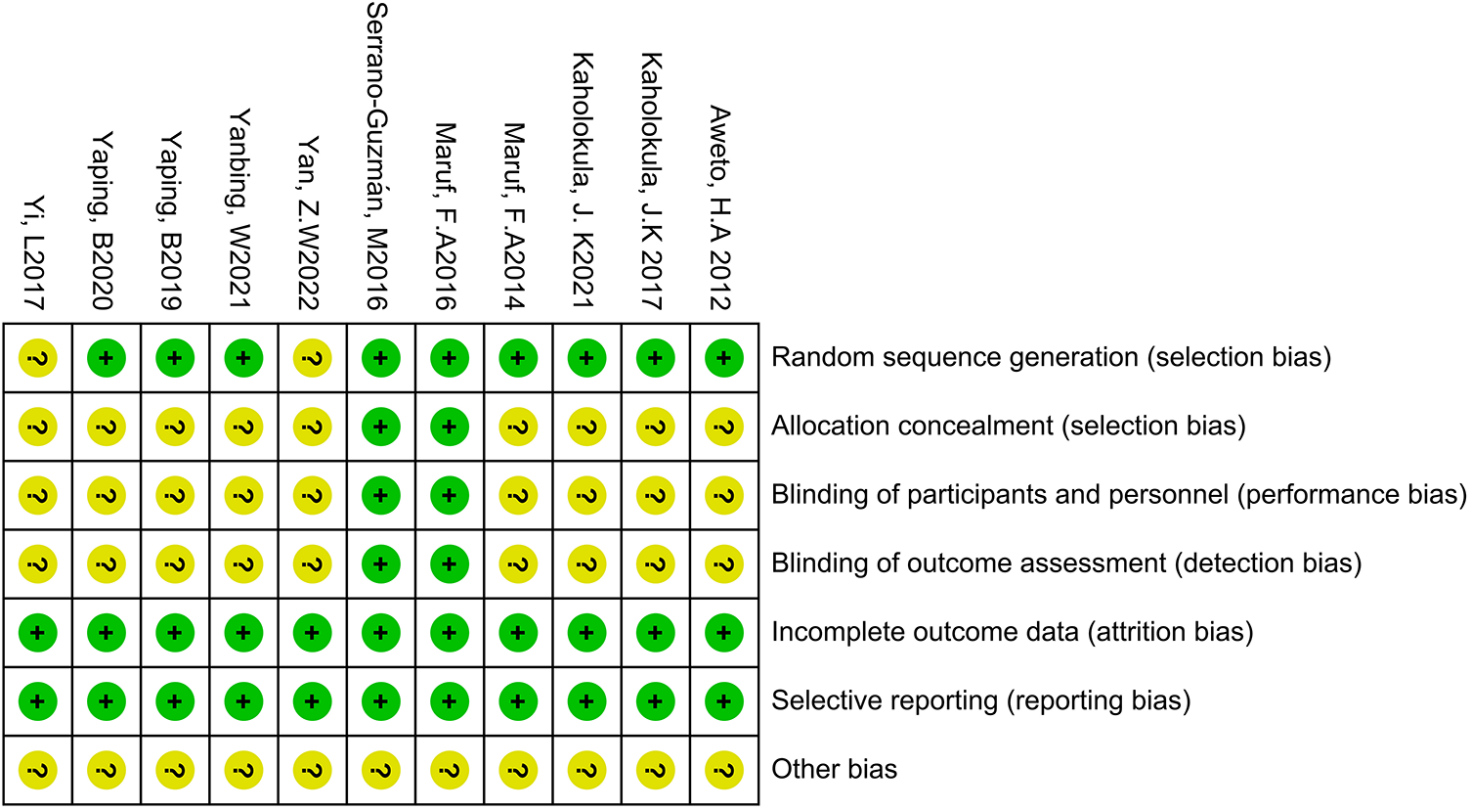
Punctuation of risk of bias tool.

The quality of the 11 included studies was assessed using the Cochrane Collaboration Tool. The results are presented in Figures 2, 3. All studies underwent randomization grouping. According to the PEDro score, all included studies were of high quality, with an average score of 6.36 points. The included studies generally met high quality standards. Supplementary Table S3 presents a detailed summary of the methodological quality assessment, including individual PEDro scores for each study.

### 3.4 Meta-analysis results

#### 3.4.1 Effects of DT on SBP

**Figure 4:**
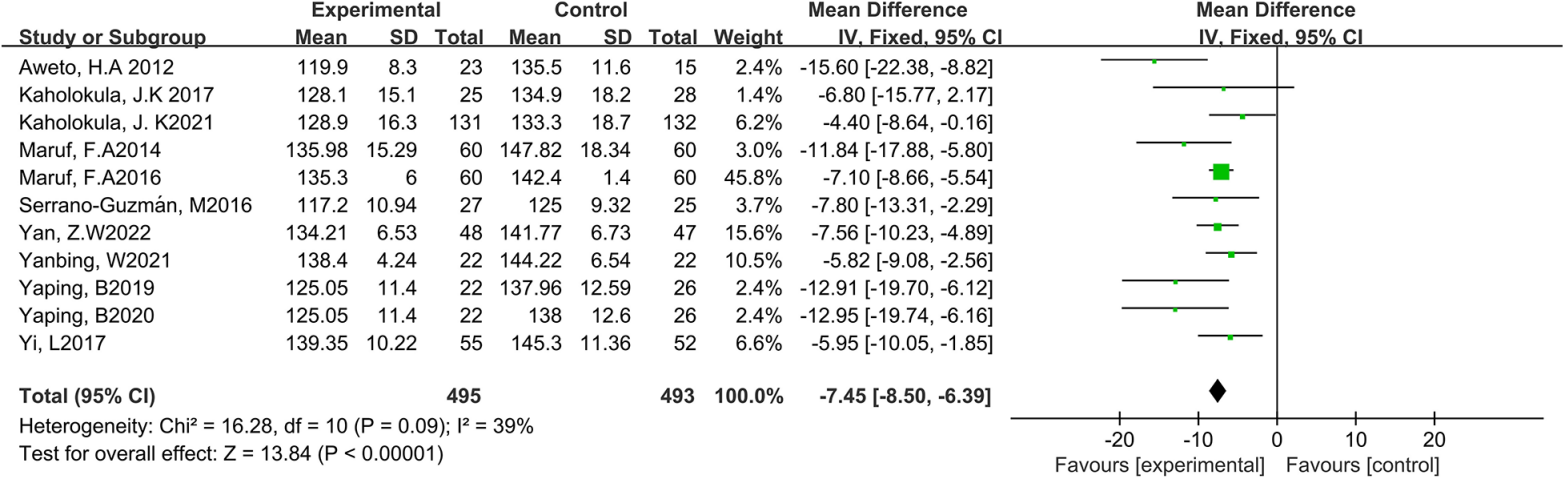
Forest plot of meta-analysis on the effect of dance on SBP.

Eleven studies reported the effect of DT on SBP (7, 12, 19–27). Due to the low heterogeneity among the included studies (*p* = 0.09, *I*^2^ = 39%), the fixed-effect model was employed for analysis. The results showed a statistically significant reduction in SBP through DT intervention [MD = −7.45, 95% CI (−8.50, −6.39), *p* < 0.0001]. As shown in Figure 4.

#### 3.4.2 Effects of DT on DBP

**Figure 5:**
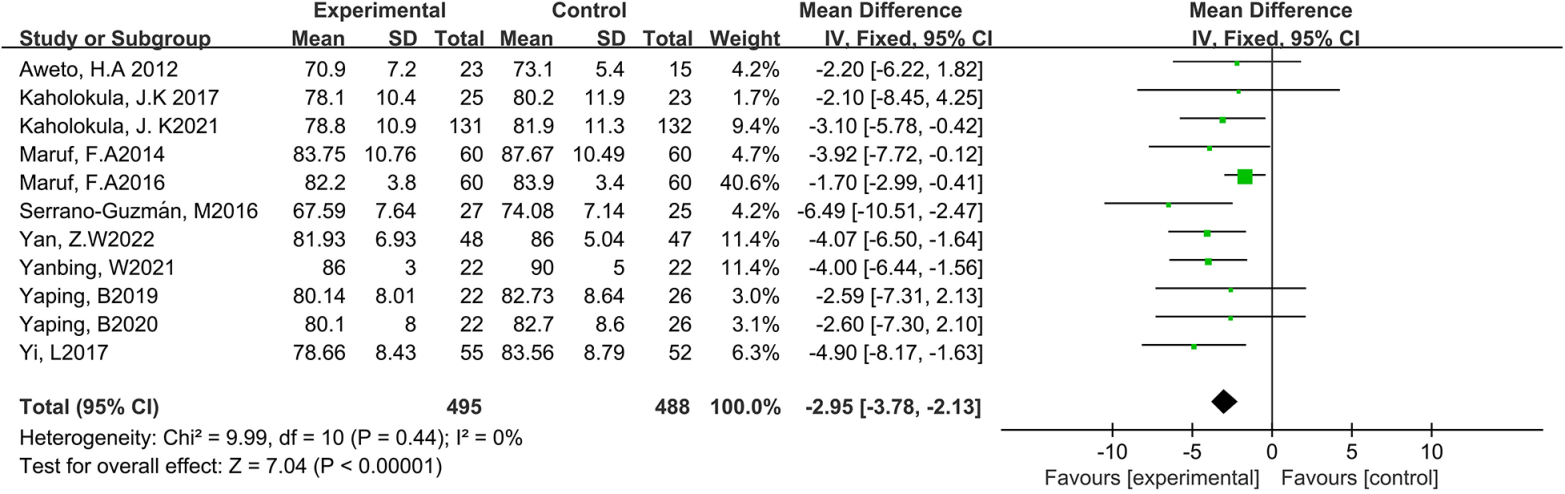
Forest plot of meta-analysis on the effect of dance on DBP.

Eleven studies reported the effect of DT on DBP (7, 12, 19–27). Due to the low heterogeneity among the included studies (*p* = 0.44, *I*^2^ = 10%), the fixed-effect model was employed for analysis. The results showed a statistically significant reduction in DBP through DT intervention [MD = −2.95, 95% CI (−3.78, −2.13), *p* < 0.0001]. As shown in Figure 5.

#### 3.4.3 Effects of DT on TC, TG, HDL-C and LDL-C

Four studies reported the effect of DT on TC, TG, HDL-C and LDL-C (20, 22, 23, 26). The heterogeneity among the included studies respectively was TC (*p* = 0.06, *I*^2^ = 59%), TG (*p* = 0.32, *I*^2^ = 14%), HDL-C (*p* = 0.30, *I*^2^ = 17%) and LDL-C (*p* = 0.57, *I*^2^ = 0%). Due to the high heterogeneity among the included studies for TC, the random-effect model was employed for analysis. The results indicated that DT did not significantly reduce TC levels [MD = −0.11, 95% CI (−0.47, 0.25), *p* = 0.55]. Due to the low heterogeneity among the included studies for TG, HDL-C and LDL-C, the fixed-effect model was employed for analysis. The results revealed that DT significantly improved HDL-C levels in hypertensive patients [MD = 0.20, 95% CI (−0.02, 0.42), *p* < 0.0001]. However, DT did not have a significant effect on TG and LDL-C levels in hypertensive patients. As shown in Table 2.

**Table 2:** Meta-analysis on the effect of dance on TC, TG, HDL-C, LDL-C.

Index	K	Sample size	Homogeneity test			Effect size	95% CI	Two-tailed test	
			*C* ^2^	*p*	*I* ^2^			*Z*	*p*
TC	4	319	7.30	0.06	59%	−0.11	−0.47,0.25	0.60	0.55
TG			3.48	0.32	14%	−0.18	−0.40,0.05	1.56	0.12
HDL-C			3.62	030	17%	0.20	−0.02,0.42	1.75	0.08
LDL-C			2.03	0.57	0%	0.01	−0.21,0.23	0.06	0.95

Note: K, number of studies in subgroup.

#### 3.4.4 Effects of DT on BMI and RHR

**Figure 6:**
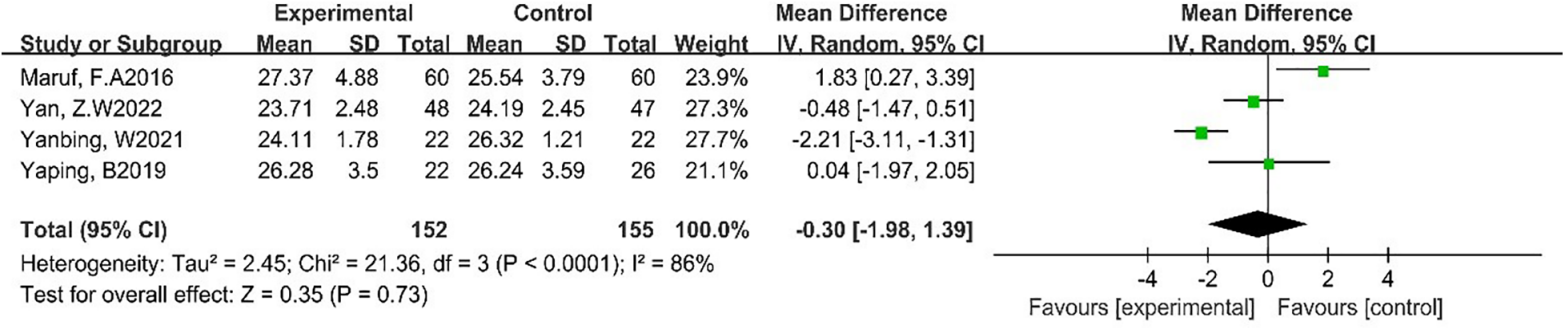
Forest plot of meta-analysis on the effect of dance on BMI.

Four studies reported the effect of DT on BMI (7, 23, 26, 27). Due to the high heterogeneity among the included studies (*p* < 0.0001, *I*^2^ = 86%), the random-effect model was employed for analysis. The results showed that DT did not significantly reduce BMI [MD = −0.30, 95% CI (−1.98, 1.39), *p* = 0.73]. As shown in Figure 6.

**Figure 7:**
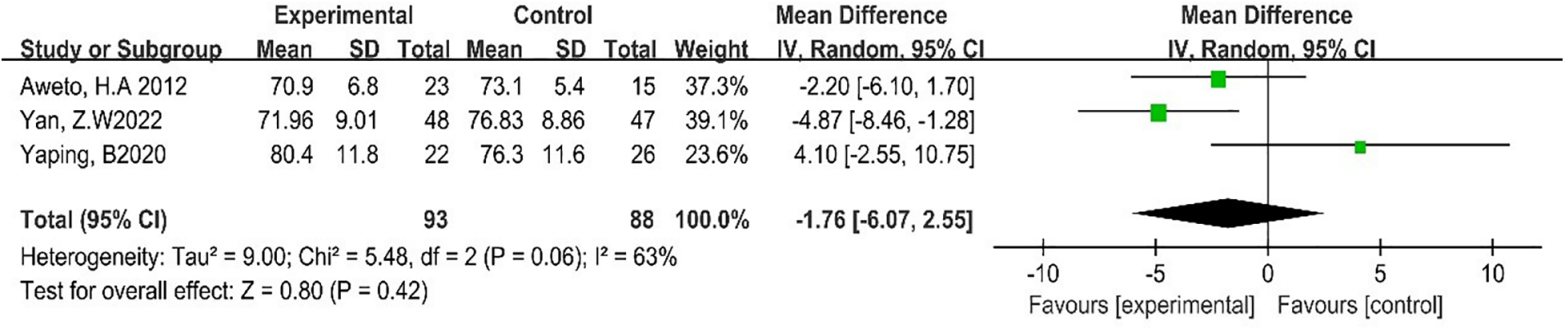
Forest plot of meta-analysis on the effect of dance on RHR.

Three studies reported the effect of DT on RHR (19, 24, 27). Due to the high heterogeneity among the included studies (*p* = 0.06, *I*^2^ = 63%), the random-effect model was employed for analysis. The results showed that DT did not significantly improve RHR [MD = −1.76, 95% CI (−6.07, 10.75), *p* = 0.42]. As shown in Figure 7.

#### 3.4.5 Subgroup analysis

Considering that exercise cycle, frequency and duration may impact blood pressure improvement in hypertensive patients, we conducted a subgroup analysis. The subgroup analysis results indicated that DT significantly reduced SBP and DBP levels in hypertensive patients when the exercise cycle was less than 12 weeks, the frequency was more than 3 times per week, and each session lasted less than 60 min. As shown in Supplementary Table S4.

#### 3.4.6 Assessment of publication bias

Egger's test was employed to assess publication bias in SBP and DBP outcome indicators. The results showed that both SBP (*t* = −1.76, *p* = 0.112) and DBP (*t* = −1.90, *p* = 0.089) had *p*-values greater than 0.05, suggesting no significant publication bias. As shown in Supplementary Figures S1, S2.

#### 3.4.7 GRADE quality evaluation

We conducted GRADE quality evaluation for the key outcome indicators of the included studies, included SBP, DBP, TC, TG, LDL-C, and HDL-C. GRADE system categorizes evidence quality into four levels: high, medium, low, and very low. It evaluates evidence based on five factors: risk of bias, inconsistency, imprecision, indirectness, and publication bias. The results are presented in Table 3.

**Table 3:** GRADE quality of evidence of the included studies.

Outcome	K	Sample size	Limitations	Inconsistency	Indirectness	Imprecision	Publication bias	Quality of the evidence
SBP	10	720	−1^①^	0	0	0	0	Moderate ⊕⊕⊕○
DBP	10	720	−1^①^	0	0	0	0	Moderate ⊕⊕⊕○
TC	4	319	−1^①^	−1^②^	0	−2^③^	0	Very Low ⊕○○○
TG	4	319	−1^①^	0	0	−1^③^	0	Low ⊕⊕○○
LDL-C	4	319	−1^①^	0	0	−1^③^	0	Low ⊕⊕○○
HDL-C	4	319	−1^①^	0	0	−1^③^	0	Low ⊕⊕○○

Notes: K, number of studies in subgroup. ①Failure to report specific random methods, allocation concealment, blinding, and loss to follow-up information; ②Larger point estimates between different studies, narrow confidence intervals or small overlap, small *p* value from heterogeneity test or large *I*^2^ value; ③Small sample size, wide confidence interval; ④Inclusion of original studies with small sample sizes, or all positive results, indicating a high likelihood of publication bias; ⑤Asymmetric funnel plot. 0 indicates no downgrade; −1 indicates a downgrade by 1 level.

## 4 Discussion

### 4.1 Effects of DT on blood pressure in patients with hypertension

Continuous elevation of systolic and diastolic blood pressure in patients with hypertension leads to chronic strain on the heart and blood vessels, causing the blood vessels gradual loss of elasticity and becoming stiffness, and increasing susceptibility to cardiovascular and cerebrovascular diseases, such as coronary heart disease, myocardial infarction, and stroke (28, 29). Research has shown that aerobic exercise may be highly effective in lowering blood pressure in hypertensive patients (30). Dance involves prolonged activity, engages large muscle groups, and fosters strong motivation and social cohesion. Dance exercise of appropriate intensity positively impacts cardiovascular blood circulation, enhancing myocardial contractility and output while alleviating cardiac strain, leading to reducing the burden of heart, preventing and improving cardiovascular disease (27, 31). Previous meta-analysis suggested that effective aerobic physical activity in hypertensive patients could reduce SBP by 5–10 mmHg and DBP by 1–6 mmHg (32). This meta-analysis showed a reduction in SBP by 7.65 mmHg and DBP by 2.94 mmHg following DT intervention consistent with previous research findings (32). The reduction of blood pressure plays a crucial role in the prevention of cardiovascular and cerebrovascular diseases, as a 5 mmHg decrease in SBP correlates with a 13% decrease in stroke risk (33, 34).

### 4.2 Effects of DT on blood lipid in patients with hypertension

Hypertension is frequently associated with dyslipidemia (35). Dyslipidemia impacts cell membrane lipid composition, leading to disrupting Ca2+ transport, damaging the vascular endothelium, promoting vascular smooth muscle cell hypertrophy, and inducing arterial structural alterations (36, 37). Arterial structural abnormalities and stiffening impede blood flow, exacerbating dyslipidemia and initiating a vicious cycle of elevated blood pressure and aberrant lipid metabolism (38, 39). Therefore, controlling lipid levels is crucial for managing blood pressure in hypertensive patients. This meta-analysis revealed improvements in TC, TG, and HDL-C following DT intervention compared to the controls, with the most significant improvement seen in HDL-C, but not in LDL-C. Long-term aerobic exercise promotes fat utilization for energy, leading to gradual normalization of lipid metabolism and blood sugar levels (40). HDL-C, an anti-atherosclerotic lipoprotein, acts as a protective factor against coronary atherosclerotic heart disease, facilitating cholesterol clearance and prevention of atherosclerosis and coronary heart disease (41). Moreover, HDL-C possesses anti-inflammatory and antioxidant properties, safeguarding vascular endothelial cells from damage, so HDL-C levels are important for lipid metabolism in hypertensive patients (42). Regular exercise can enhance lipoprotein lipase activity and the skeletal muscle's ability to utilize fatty acids for energy, thereby promoting lipid metabolism (43). Exercise may also regulate the synthesis, transport, and catabolism of lipoproteins by altering the activities of lecithin-cholesterol acyltransferase (LCAT), lipoprotein lipase (LPL), and hepatic triglyceride lipase (HTGL) (44). Additionally, exercise can induce lipolysis of lipids in adipose tissue and muscle, transporting fatty acids to muscles and regulating transmembrane transport and mitochondrial metabolism of fatty acids in muscle cells, ultimately improving lipid metabolism and lowering TC, TG, and LDL-C levels in patients with hypertension (45).

### 4.3 Effects of DT on BMI and RHR in patients with hypertension

With the improvement of living standards, the prevalence of obesity has been increasing annually (46). Excess body fat significantly influences the occurrence and development of cardiovascular diseases such as hypertension (47, 48). obesity can lead to metabolic disorders and increase the risk of cardiovascular diseases, thus increasing the incidence and mortality of hypertension (49). Heart rate, a clinical indicator for assessing sympathetic nervous system activity, is frequently employed to predict the prognosis of cardiovascular diseases such as hypertension (50). Patients with hypertension generally exhibit heightened sympathetic nervous system activity, contributing to elevated heart rates. Long-term maintenance of this state will lead to increased blood vessel pressure, aggravate hypertension, and precipitate severe conditions such as myocardial infarction, posing a life-threatening risk to patients (51, 52). This meta-analysis showed that BMI and RHR improved after the dance therapy intervention, however, the improvements were not statistically significant relative to the control group (*p* > 0.05). Dance can control the body mass of hypertensive patients, enhance cardiac contractility and muscle endurance, modulate vagal tone and diminish sympathetic nervous system activity (53). This enhances the efficiency of blood pumping by the heart, thereby reducing the heart rate (54).

## 5 Conclusion

Dance, as a group activity, fosters integration within hypertensive patients’ communities, rebuilds social relationships and preserves youthful vitality. In addition, dance exercise can also relieve stress, alleviate anxiety and promote physical and mental relaxation (55). Through enhancing the function of the human endocrine system, dance can regulate emotions and psychological states, thereby releasing personal pressure (56). It significantly benefits the physical and mental health of hypertensive patients and improves the treatment effects of susceptible diseases in middle-aged and elderly people (24).

This systematic review and meta-analysis provided evidence indicating a significant reduction in SBP and DBP following dance therapy. When the exercise cycle was less than 12 weeks, the exercise frequency was more than 3 times per week, and the duration of each exercise was less than 60 min, the levels of SBP and DBP decreased greatly. Therefore, it is recommended that patients with hypertension perform DT for 12 weeks as an exercise cycle, with sessions exceeding 3 times weekly, each lasting within 60 min. At present, there lacks a standardized exercise prescription for dance therapy. It is necessary for further research to provide additional clinical evidence for the application and promotion of dance therapy.

## 6 Limitation

(1) This systematic review and meta-analysis included only 11 studies, predominantly from Chinese sources, and the difference in studies quality might influence the results.
(2) None of the studies we included provided post-intervention follow-up.
(3) High heterogeneity among some included studies may affect the reliability of the results of meta-analysis.
(4) Differences in exercise prescription designs for dance therapy within the included studies may influence the intervention effect to some extent and may affect the reliability of the meta-analysis results.

## Data Availability

The original contributions presented in the study are included in the article/Supplementary Material, further inquiries can be directed to the corresponding author.
