# Supplementary material for: Does dance therapy benefit the improvement of blood pressure and blood lipid in patients with hypertension? A systematic review and meta-analysis
Source: Front Cardiovasc Med. 2024 Oct 24;11:1421124. doi: 10.3389/fcvm.2024.1421124 (PMC11540642; doi:10.3389/fcvm.2024.1421124)

Supplementary Table 1.Search strategy for each database

Search engine	Search query	Date of search
CNKI/VIP/ Wanfang Data /CBM	#1 主题 (Topic) =舞蹈(dance) OR 舞蹈训练(dance training) OR 舞蹈锻炼(dancing exercises) OR 有氧舞蹈(aerobic dance)<br>#2 主题 (Topic)= 高血压(hypertension/high blood pressure) OR 血压(blood pressure)<br>#5 #1 AND #2	January 10, 2024
Pubmed	#1 dance [MeSH Major Topic]<br>#2 dance training [Text Word]<br>#3 dancing exercises [Text Word]<br>#4 aerobic dance [Text Word]<br>#5 #1 OR #2 OR #3 OR #4<br>#6 hypertension [MeSH Major Topic]<br>#7 high blood pressure [Text Word]<br>#8 blood pressure [Text Word]<br>#9 #6 OR #7 OR #8<br>#10 #5 AND #15	January 10, 2024
Web of science	#1 TS = (‘dance’ OR ‘dance training’ OR ‘dancing exercises’ OR ‘aerobic dance’)<br>#2 TS = (‘hypertension’ OR ‘high blood pressure’ OR ‘blood pressure’)<br>#3 #1 AND #2<br>Databases = SCI-EXPANDED, SSCI, A&HCI, CPCI-S, CPCI-SSH, ESCI	January 10, 2024
Embase	#1 ‘dance’ OR ‘dance training’ OR ‘dancing exercises’ OR ‘aerobic dance’<br>#2 ‘hypertension’ OR ‘high blood pressure’ OR ‘blood pressure’<br>#3 #1 AND #2	January 10, 2024
Cochrane Library	S1 dance OR dance training OR dancing exercises OR aerobic dance<br>S2 whole-body vibration training OR vibration training OR vibration OR VT OR WBVT<br>S3 S1 AND S2	January 10, 2024

Table note: CNKI: China National Knowledge Infrastructure; VIP:VIP database; CBM: China Biology Medicine disc

Supplementary Table 2 Description of intervention measures

Intervention	Definition
Square dance	The type of aerobic fitness activity that is primarily performed in public squares where people gather to participate.
Taiji softball dance	This approach combines Taiji softball with dance, incorporating fundamental techniques of Taiji softball and fitness dance movements.
Dance therapy	The dance therapy is based on Spanish folk dance (flamenco and sevillanas).
Hula	The hula classes involved instructions in dance footwork, upper body movements, and learning poetry, stories, history, Hawaiian language, and meaning of the accompanying songs and chants.
Aerobic dance	The aerobic dance incorporated bouncing movement on the leg, forward and backward movement, jumping, shoulder movements, arm stretching, trunk stretching, rolling and bending of the trunk, hip twisting, and chest movement.

Supplementary Table 3 PEDro scale score of included literature

Reference	PEDro scale											
	①	②	③	④	⑤	⑥	⑦	⑧	⑨	⑩	⑪	score
Yaping, B2019	Y	1		1				1	1	1	1	6
Yaping, B2020	Y	1		1				1	1	1	1	6
Yanbing, W2021	Y	1		1				1	1	1	1	6
Yi, L2017	Y	1		1				1	1	1	1	6
Yan, Z.W2022	Y	1		1				1	1	1	1	6
Aweto, H.A 2012	Y	1		1				1	1	1	1	6
Kaholokula, J.K 2017	Y	1		1				1	1	1	1	6
Maruf, F.A2014	Y	1		1				1	1	1	1	6
Serrano-Guzmán, M2016	Y	1	1	1	1			1	1	1	1	8
Maruf, F.A2016	Y	1	1	1	1			1	1	1	1	8
Kaholokula, J. K	Y	1		1				1	1	1	1	6

Note:①Whether the inclusion conditions of subjects are clear;②Randomly grouping;③Blind grouping; ④Baseline consistency of main prognostic indicators;⑤The subjects were blinded;⑥Training is blind; ⑦All the evaluators of at least one main result are blind;⑧Measure at least one main result for more than 85% of the subjects;⑨Assign subjects according to the distribution scheme to receive treatment or control conditions;⑩Report at least one main result of inter-group statistical results;⑪Provide at least one point measurement and variation measurement of the main result.

Supplementary Table 4 The subgroup analysis of blood pressure

Index	Variable	Group	K	Sample size	Homogeneity test			Effect size and 95% CI	Two-tailed test	
					C^2	P	I^2		Z	P
SBP	Duration	≥12W	8	773	10.05	0.16	33%	-7.34 [-8.86, -5.82]	9.46	0.00
		<12W	3	210	5.74	0.06	65%	-9.27 [-13.67,-4.87]	4.13	0.00
	Frequency	>3/W	5	547	8.52	0.07	53%	-7.07 [-9.61, -4.53]	5.45	0.00
		≤3/W	6	436	6.91	0.24	28%	-7.86 [-9.24,-6.49]	11.18	0.00
	Time	≥60min/d	7	653	8.23	0.22	27%	-7.04 [-8.61, -5.47]	8.77	0.00
		< 60min/d	4	330	7.58	0.06	60%	-9,70[-13.39,-6.01]	5.15	0.00
DBP	Duration	≥12W	8	773	1.54	0.98	0%	-3.72 [-4.89, -2.55]	6.25	0.00
		<12W	3	210	4.95	0.08	60%	-3.04[-5.79,-0.29]	2.17	0.03
	Frequency	>3/W	5	547	1.38	0.85	0%	-3.78 [-5.05, -2.50]	5.79	0.00
		≤3/W	6	436	5.74	0.33	13%	-2.34 [-3.43,-1.25]	4.22	0.00
	Time	≥60min/d	7	653	1.53	0.96	0%	-3.70 [-4.93, -2.47]	5.92	0.00
		< 60min/d	4	330	5.71	0.13	47%	-2.31 [-3.43,-1.18]	4.03	0.00

Table Notes: K, number of studies in subgroup.

Supplementary Figure 1 SBP publication bias graph of the included literature

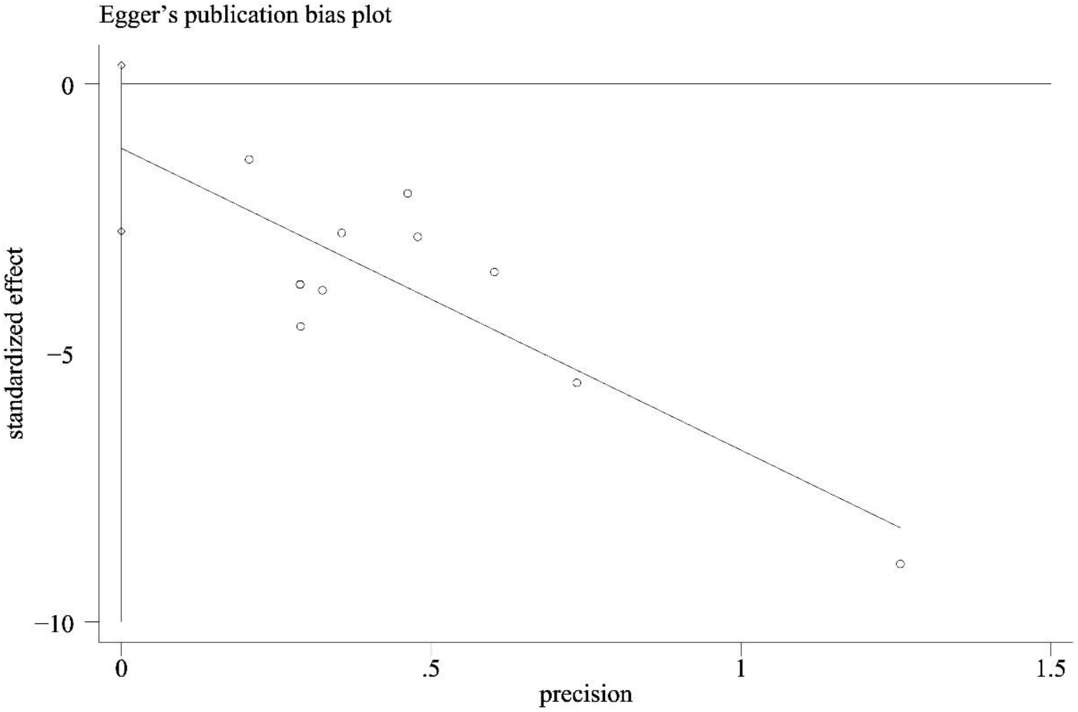

Supplementary Figure 2 DBP publication bias graph of the included literature

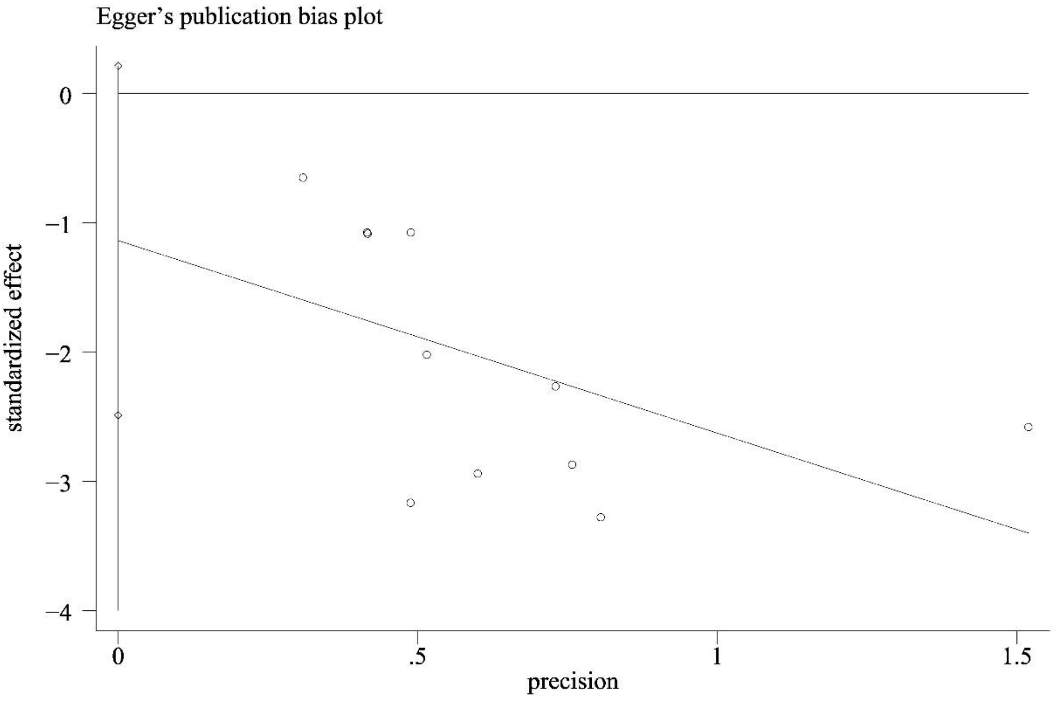

Supplement: Supplementary file 1 [file Datasheet1.pdf]
